# Marginal improvement in survival among patients diagnosed with metastatic prostate cancer in the second‐line antiandrogen therapy era

**DOI:** 10.1002/cam4.4074

**Published:** 2021-10-29

**Authors:** Isaac E. Kim, Thomas L. Jang, Sinae Kim, David Y. Lee, Daniel D. Kim, Eric A. Singer, Saum Ghodoussipour, Mark N. Stein, Monish Aron, Marc A. Dall’Era, Isaac Yi Kim

**Affiliations:** ^1^ Warren Alpert Medical School Brown University Providence RI USA; ^2^ Section of Urologic Oncology Rutgers Cancer Institute of New Jersey and Division of Urology Rutgers Robert Wood Johnson Medical School The State University of New Jersey New Brunswick NJ USA; ^3^ Department of Biostatistics and Epidemiology Rutgers School of Public Health The State University of New Jersey Piscataway NJ USA; ^4^ Department of Internal Medicine Division of Medical Oncology Columbia University New York NY USA; ^5^ Department of Urology University of Southern California Los Angeles CA USA; ^6^ Department of Urologic Surgery University of California Davis Sacramento CA USA

**Keywords:** metastatic prostate cancer, M1 prostate cancer, second‐line antiandrogens, survival

## Abstract

Since 2004, multiple blockbuster drugs have been approved for men with metastatic prostate cancer. Nevertheless, it has been reported that no improvement in survival was observed between 2004 and 2009. Herein, we have analyzed the SEER database to assess the survival outcome of metastatic prostate cancer patients since 2000. The results demonstrated that there was an improvement in both overall and prostate cancer‐specific survival for 4 months among men diagnosed with metastatic prostate cancer from 2010 to 2016 when compared to those in the pre‐2010 period. Interestingly, this survival benefit was limited to patients with bone and visceral metastasis (M1b and M1c stages). Collectively, our observation suggests that despite the new treatment agents such as second‐line antiandrogen therapies introduced in the modern era, the improvement in survival of metastatic prostate cancer patients has been surprisingly small.

## Graphical abstract

Since 2000–2003, there has been an improvement in both overall and cancer‐specific survival of only 4 months in men diagnosed with metastatic prostate cancer.

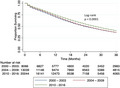

## 1 INTRODUCTION

Prostate cancer is the second most frequently diagnosed cancer in men and the fifth leading cause of death with 1,276,106 new cases and 358,989 deaths worldwide in 2018 alone.^1^ At some point during their lives, an estimated 12.1% of men will be diagnosed with prostate cancer. In 2017, 3,170,339 men were living with prostate cancer in the United States. On presentation, 76% are localized or confined to the prostate, 13% have spread to regional lymph nodes, 6% have metastasized, and 5% are unknown or unstaged.^2^ This distribution is significant in which survival rates widely vary depending on stage with 5‐year relative survival rates for localized, regional, and metastatic prostate cancer being approximately 100%, 100%, and 31%, respectively.^3^

In men diagnosed with metastatic disease, androgen deprivation therapy (ADT) is the initial standard of care.^4^ However, prostate cancer cells eventually become resistant to ADT and castration‐resistant prostate cancer (CRPC) emerges. In men with CRPC, broadly available treatment options are chemotherapy, immunotherapy, and second‐line antiandrogen therapy (SAT).^5^ In 2004, the USFDA approved docetaxel, the first agent shown to improve survival rates in patients with CRPC.^6^ Since then, several drugs shown to improve survival rates have been approved including cabazitaxel and sipuleucel‐T in 2010 and second‐line antiandrogen therapies (SAT) abiraterone and enzalutamide in 2011 and 2012, respectively.^7^ More recently, additional SAT agents, apalutamide and darolutamide, as well as the PARP inhibitor olaparib have been added to the armamentarium against CRPC.^8^, ^9^, ^10^

Despite the approval of docetaxel in 2004, Wu and colleagues reported that overall and prostate cancer‐specific survival did not improve in patients with metastatic disease in the docetaxel era (years 2004–2009).^11^ However, given the recent advent of several blockbuster drugs in the SAT class for CRPC since 2010 combined with the implementation of more intense therapy supported by clinical trials,^12^, ^13^ we hypothesized that there would be significant improvements in survival among patients diagnosed with metastatic prostate cancer from the pre‐docetaxel to SAT era (years 2000–2016).

## 2 MATERIALS AND METHODS

### 2.1 Data Sources

The study sample was composed of patients at least 18 years old with distant prostate cancer from the Surveillance, Epidemiology, and End Results (SEER) Program, which publishes epidemiologic data on the incidence and survival rates of cancer in the United States. Using SEER*Stat statistical software, patient information was extracted from the Incidence – SEER 18 Regs Research Data, Nov 2018 Sub (2000–2016), which collects information on patients from Connecticut, Detroit, Atlanta, San Francisco‐Oakland, Hawaii, Iowa, New Mexico, Seattle‐Puget Sound, and Utah, San Jose‐Monterey, Los Angeles, Alaska Native Registry, Rural Georgia, California excluding San Francisco/San Jose‐Monterey/Los Angeles, Kentucky, Louisiana, New Jersey, and George excluding Atlanta/Rural Georgia. Information on chemotherapy and radiation treatment for these patients was extracted from Incidence – SEER 18 Regs Custom Data (with additional treatment fields), Nov 2018 Sub (2000–2016).

### 2.2 Study variables

This study primarily examined the following study variables: age, race/ethnicity, year of diagnosis, treatment with prostatectomy and/or radiotherapy or no local therapy, and metastatic subclass. Metastatic subclass was based on the American Joint Committee on Cancer (AJCC) Stage 3^rd^ edition for patients diagnosed in 2000–2003, adjusted AJCC 6^th^ edition for those in 2004–2015, and derived SEER Combined Stage M for those in 2016.

### 2.3 Statistical analysis

The primary study outcomes were overall survival (OS) and prostate cancer‐specific survival (PCSS) based on the diagnostic time period and metastatic subclass when available. Secondary study outcomes investigated changes across years in the distribution of age, race/ethnicity, and treatment with prostatectomy and/or radiotherapy or no local therapy.

The following three time periods were examined: January 2000–December 2003 (era 1), January 2004–December 2009 (era 2), and January 2010–December 2016 (era 3). Overall and prostate cancer‐specific survival were estimated using the Kaplan–Meier product limit method stratified by three time periods and their differences were analyzed using the log‐rank test and univariate and multivariable Cox proportional hazards models were applied to assess the risk of death and prostate cancer‐specific death with time periods. Hazard ratios (HR) and 95% confidence intervals were reported. In multivariable model, age, treatment, and race/ethnicity were analyzed. Because stage is not available for era 1, the stage was adjusted in comparing eras 2 and 3. Changes in the distribution of age, race/ethnicity, and treatment across time periods were analyzed using Pearson's chi‐square test. All statistical analyses were performed using Stata/SE 15.0 with a *p*‐value of less than or equal to 0.05 considered statistically significant.

## 3 RESULTS

### 3.1 Overall survival of patients with metastatic prostate cancer shows modest improvements between 2000 and 2016

The study sample consisted of 41,149 patients at least 18 years old diagnosed with distant prostate cancer between 2000 and 2016. We excluded 392 patients from era 1 (years 2000–2003), 159 from era 2 (years 2004–2009), and 192 from era 3 (years 2010–2016) for being M0 due to the likelihood of stage misattribution. Median follow‐up for era 1, 2, and 3 for OS was 179, 117, and 36 months, respectively.

Patient characteristics among the three eras are shown in Table 1. Among the variables examined, men in the most recent SAT era (era 3, 2010–2016) were younger with a median age of 71 versus 73 and 72 in era 1 and era 2, respectively (*p *< 0.001). The racial/ethnic distribution of patients with metastatic prostate cancer also changed with the number of non‐Hispanic white and black patients decreasing from 64.59% in 2000–2003 to 63.13% in 2010–2016 and 19.89% to 17.87%, respectively, and the number of Hispanic, non‐Hispanic Asian/Pacific Islander, and non‐Hispanic American Indians/Alaskan Natives increasing from 9.63% to 11.91%, 5.22% to 5.78%, and 0.43% to 0.60% during that same time period, respectively (*p *< 0.001). In addition, a stage migration was observed between eras 2 and 3 with a significant shift from M1c to M1b and M1a (*p *< 0.001). Breakdown of metastatic stage and serum prostate‐specific antigen levels was not available for era 1. Notably, there was no significant change in the utilization of local treatment (*p *= 0.376).

**TABLE 1:** Characteristics of patients with metastatic prostate cancer

	Era 1, 2000–2003	Era 2, 2004–2009	Era 3, 2010–2016	*p*‐value
Sample size	8,066	13,039	20,044	
Median age (years) (range)	73 (19–99)	72 (28–99)	71 (18–99)	< 0.001
Median follow‐up (months) (range)	179 (177–180)	117 (115–118)	36 (35–36)	
Median overall survival (months)	24 (23–25)	24 (24–25)	28 (27–28)	< 0.001
Median cause‐specific survival (months)	24 (23–24)	24 (24–25)	28 (27–28)	<0.001
Race/ethnicity				< 0.001
Non‐Hispanic White	5,210 (64.59%)	8,356 (64.08%)	12,653 (63.13%)	
Non‐Hispanic Black	1,604 (19.89%)	2,366 (18.15%)	3,582 (17.87%)	
Hispanic	777 (9.63%)	1,448 (11.11%)	2,388 (11.91%)	
Non‐Hispanic Asian/PI	421 (5.22%)	756 (5.80%)	1,159 (5.78%)	
Non‐Hispanic American Indian/Alaska Native	35 (0.43%)	78 (0.60%)	142 (0.71%)	
Non‐Hispanic Unknown Race	19 (0.24%)	35 (0.27%)	120 (0.60%)	
Stage				< 0.001
Blank(s) or N/A	6 (0.07%)	29 (0.22%)	95 (0.47%)	
M1NOS	‐	488 (3.74%)	1,125 (5.61%)	
M1	8,059 (99.91%)	12,492 (95.80%)	18,819 (93.89%)	< 0.001
M1a	‐	627 (5.02%)	1,165 (6.19%)	
M1b	‐	8,537 (68.34%)	14,078 (74.81%)	
M1c	‐	3,328 (26.64%)	3,576 (19.00%)	
MX	1 (0.01%)	30 (0.23%)	5 (0.02%)	
Local Treatment				0.376
Prostatectomy and/or Radiotherapy	170 (2.11%)	313 (2.40%)	466 (2.32%)	
No local therapy	7,896 (97.89%)	12,726 (97.60%)	19,578 (97.68%)	

**FIGURE 1:**
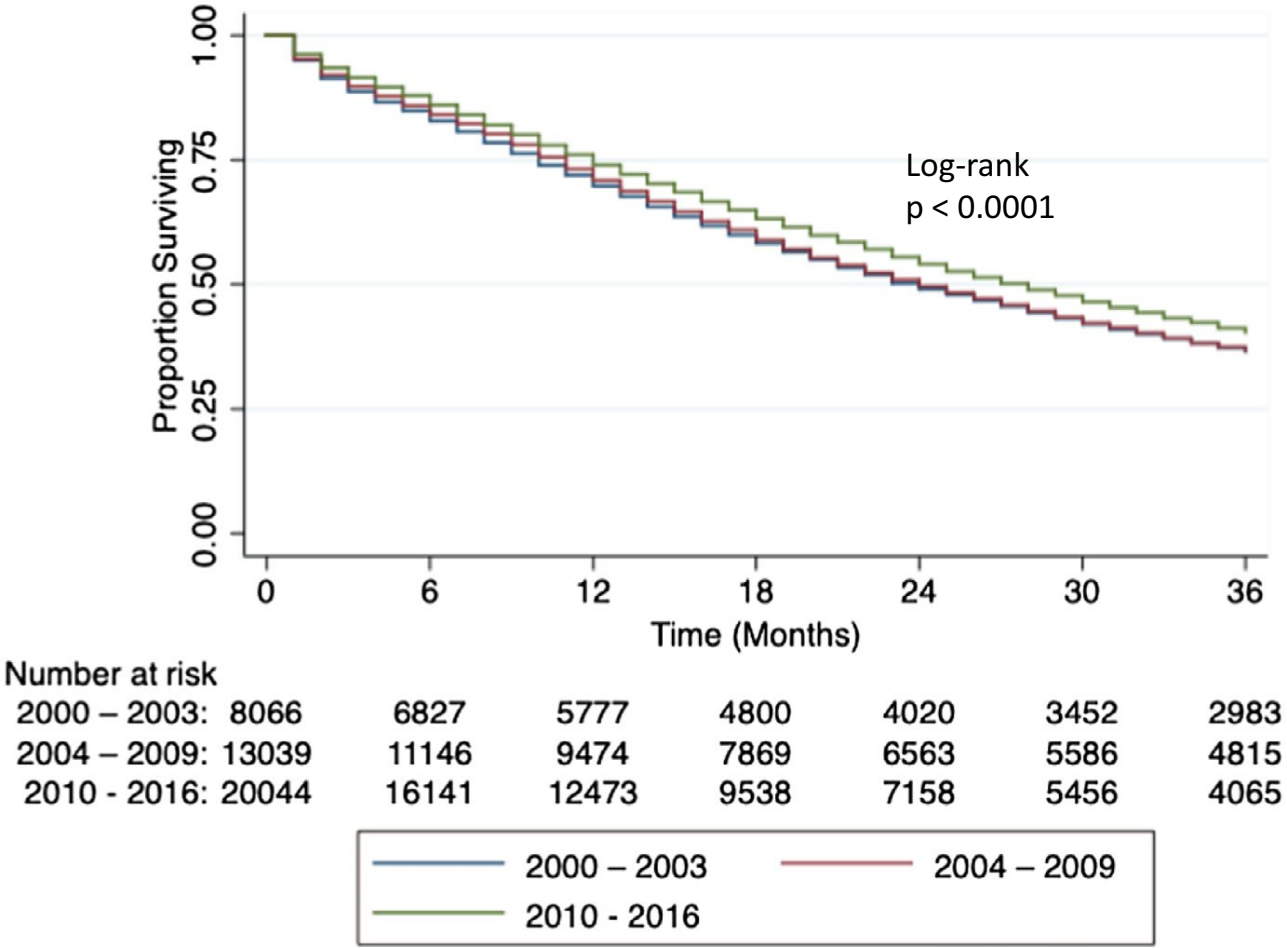
Three‐year overall survival of patients with metastatic prostate cancer in 2000–2003, 2004–2009, and 2010–2016. Overall survival of patients with metastatic prostate cancer did not change from 2000–2003 to 2004–2009 but improved from 2004–2009 to 2010–2016

The differences in OS across the three time periods were statistically significant. Median OS was 24 months in both eras 1 and 2, while OS increased by 4 months to 28 months in era 3 (*p *< 0.0001). Kaplan–Meier curve for 3‐year OS is shown in Figure 1 (*p *< 0.0001, log‐rank test). Using era 1 as the referent in Cox proportional hazard model (Table 2), era 2 was not associated with OS in both the unadjusted (HR 0.9858, 95% CI 0.9519–1.0209, *p *= 0.4233) and adjusted analysis (HR 1.0078, 95% CI 0.9731–1.0437, *p *= 0.6650). In contrast, era 3 was associated with improved OS unadjusted (HR 0.8795, 95% CI 0.8499–0.9102, *p *< 0.0001) and adjusted (HR 0.9182, 95% CI 0.8871–0.9503, *p *< 0.0001). In the adjusted analysis, we accounted for age, local therapy, and race/ethnicity. Additional factors correlating with OS were Hispanic ethnicity (adjusted HR 0.9496, 95% CI 0.9089–0.9921, *p *= 0.0207), Asian race (adjusted HR 0.7406, 95% CI 0.6957–0.7884, *p *< 0.0001), and receipt of local therapy (adjusted HR 0.3741, 95% CI 0.3277–0.4272, *p *< 0.0001). In contrast, non‐Hispanic Black race and age older than 70 were associated with a significantly shorter OS (adjusted HR 1.0651, 95% CI 1.0288–1.1027, *p *= 0.0004 and adjusted HR 1.6193, 95% CI 1.5303–1.7135, *p *< 0.0001, respectively).

**TABLE 2:** Cox proportional hazards analysis of factors associated with overall survival

	Sample size (%)	HR (95% CI)	p‐value	Adjusted HR (95% CI)	p‐value
Sample Size	41,149				
Era					
1 (2000–2003)	8,066 (19.60%)	1 (Referent)		1 (Referent)	
2 (2004–2009)	13,039 (31.69%)	0.9858146 (0.9519384 –1.020896)	0.4233	1.007765 (0.9730918–1.043673)	0.6650
3 (2010–2016)	20,044 (48.71%)	0.8795156 (0.8498592–0.9102069)	<0.0001	0.918208 (0.8871394–0.9503646)	<0.0001
Age					
<55	2,817 (6.85%)	1 (Referent)		1 (Referent)	
55–70	16,162 (39.28%)	0.9918329 (0.935859–1.051155)	0.7820	0.9987666 (0.9423184–1.058596)	0.9668
>70	22,170 (53.88%)	1.639387 (1.55011–1.733806)	<0.0001	1.619348 (1.530347–1.713525)	<0.0001
Race/Ethnicity					
Non‐Hispanic White	26,219 (63.72%)	1 (Referent)			
Non‐Hispanic Black	7,552 (18.35%)	0.9801204 (0.9471046–1.014287)	0.2507	1.065119 (1.028826–1.102693)	0.0004
Hispanic	4,613 (11.21%)	0.9011678 (0.8246194–0.9721516)	<0.0001	0.9495967 (0.9089019–0.9921135)	0.0207
Non‐Hispanic Asian/PI	2,336 (5.68%)	0.7530729 (0.7074133–0.8016795)	<0.0001	0.7405733 (0.6956624–0.7883835)	<0.0001
Non‐Hispanic American Indian/Alaska Native	255 (0.62%)	1.024736 (0.8701149–1.206832)	0.7697	1.056592 (0.8971218–1.244408)	0.5096
Non‐Hispanic Unknown Race	174 (0.42%)	0.2887188 (0.2029708–0.4106922)	<0.0001	0.3077151 (0.2163109–0.4377431)	<0.0001
Treatment					
No local therapy	40,200 (97.69%)	1 (Referent)			
Prostatectomy and/or Radiotherapy	949 (2.31%)	0.3235701 (0.2834962–0.3693087)	<0.0001	0.3741484 (0.3277183–0.4271566)	<0.0001

### 3.2 Prostate cancer‐specific survival (PCSS) of patients with metastatic prostate cancer also shows modest improvements between 2000 and 2016

**FIGURE 2:**
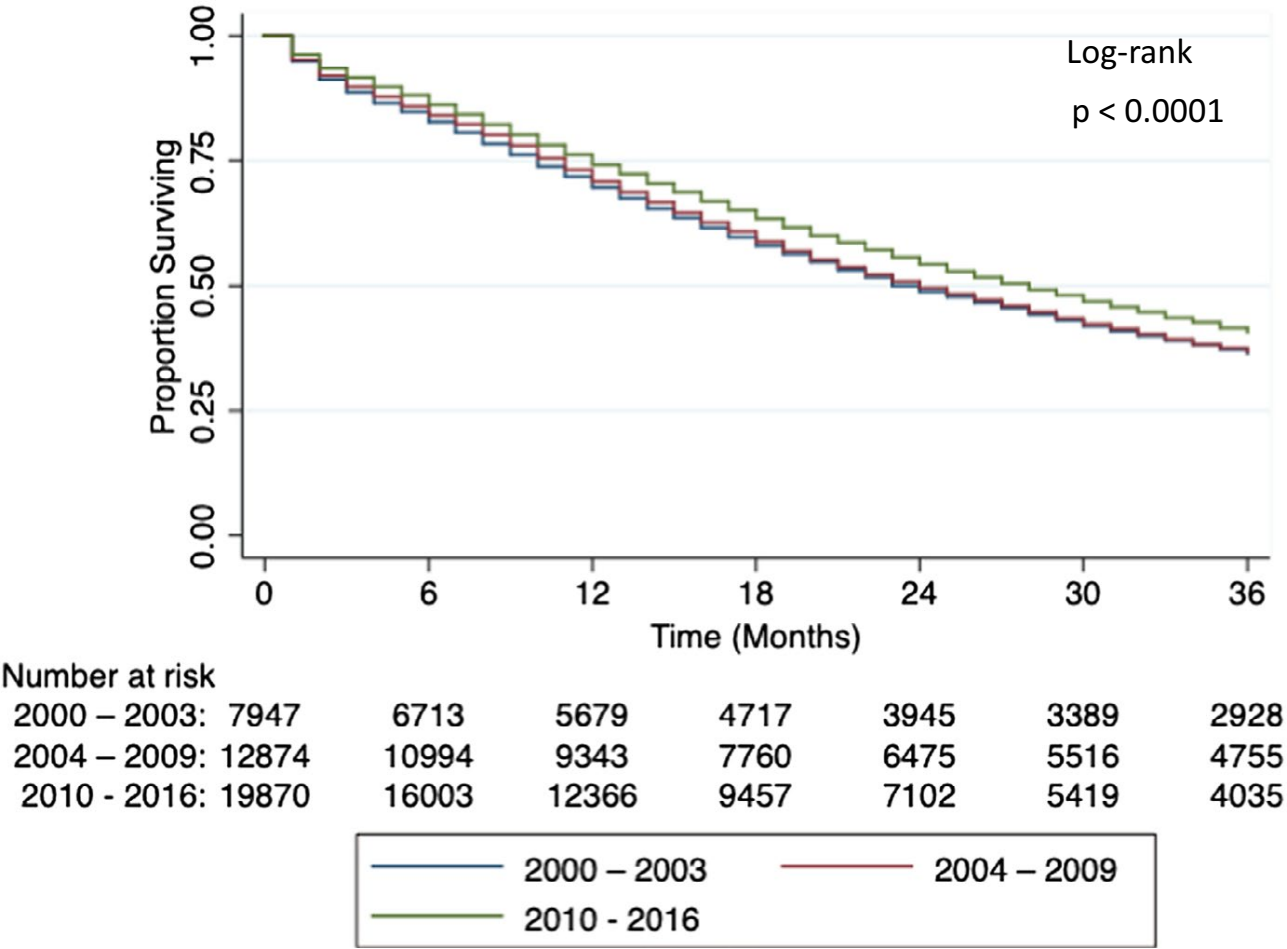
Three‐year prostate cancer‐specific survival of patients with metastatic prostate cancer in 2000–2003, 2004–2009, and 2010–2016. Prostate cancer‐specific survival of patients with metastatic prostate cancer did not change from 2000–2003 to 2004–2009 but improved from 2004–2009 to 2010–2016

As with OS, differences in cause‐specific survivals across the three time periods also were statistically significant (Table 1). The Kaplan–Meier curve for 3‐year PCSS is shown in Figure 2 (*p *< 0.0001, log‐rank test). The Cox proportional hazard model demonstrated that PCSS also did not change from era 1 to era 2 (Table 3, adjusted HR 1.0042, 95% CI 0.9694–1.0402, *p *= 0.8169) but did improve in era 3 (adjusted HR 0.9088, 95% CI 0.8778–0.9408, *p *< 0.0001). Again, adjusted variables were age, local treatment, and race/ethnicity. Additional factors associated with improved PCSS were Hispanic ethnicity (adjusted HR 0.9330, 95% CI 0.8920–0.9758, *p *= 0.0025), Asian race (adjusted HR 0.7364, 95% CI 0.6909–0.7850, *p *< 0.0001), and receipt of local treatment (adjusted HR 0.3751, 95% CI 0.3284–0.4285, *p *< 0.0001). Non‐Hispanic Black race and age older than 70 were again associated with shorter PCSS (adjusted HR 1.0652, 95% CI 1.0287–1.1029, *p *= 0.0004 and adjusted HR 1.6180, 95% CI 1.5284–1.7127, *p *< 0.0001, respectively).

**TABLE 3:** Cox proportional hazards analysis of factors associated with prostate cancer cause‐specific survival

	Sample size (%)	HR (95% CI)	p‐value	Adjusted HR (95% CI)	p‐value
Sample Size	40,691				
Era					
1 (2000–2003)	7,947 (19.53%)	1 (Referent)		1 (Referent)	
2 (2004–2009)	12,874 (31.64%)	0.9818343 (0.9478786–1.017006)	0.3073	1.004173 (0.9693995–1.040194)	0.8169
3 (2010–2016)	19,870 (48.83%)	0.8690548 (0.8395518–0.8995946)	< 0.0001	0.9087725 (0.8778082–0.940829)	< 0.0001
Age					
<55	2,786 (6.85%)	1 (Referent)			
55–70	15,979 (39.27%)	0.9881517 (0.9320063–1.047679)	0.6896	0.9957881 (0.9391212–1.055874)	0.8877
>70	21,926 (53.88%)	1.639753 (1.549871–1.734847)	< 0.0001	1.617965 (1.528446–1.712727)	< 0.0001
Race/Ethnicity					
Non‐Hispanic White	26,072 (64.07%)	1 (Referent)		1 (Referent)	
Non‐Hispanic Black	7,491 (18.41%)	0.9797775 (0.9466313–1.014084)	0.2446	1.065174 (1.028717–1.102923)	0.0004
Hispanic	4,446 (10.93%)	0.8836873 (0.8450075–0.9241375)	< 0.0001	0.9329723 (0.8919932–0.975834)	0.0025
Non‐Hispanic Asian/PI	2,266 (5.57%)	0.747698 (0.7014693–0.7969732)	< 0.0001	0.736435 (0.6908949–0.7849779)	< 0.0001
Non‐Hispanic American Indian/Alaska Native	251 (0.62%)	1.018916 (0.8636973–1.202029)	0.8241	1.052583 (0.8921905–1.241809)	0.5435
Non‐Hispanic Unknown Race	165 (0.41%)	0.2550605 (0.1736043–0.3747366)	< 0.0001	0.2702657 (0.183941–0.3971032)	< 0.0001
Treatment					
No local therapy	39,749 (97.68%)	1 (Referent)		1 (Referent)	
Prostatectomy and/or Radiotherapy	942 (2.32%)	0.3243127 (0.2839755–0.3703795)	< 0.0001	0.3751206 (0.3283706–0.4285264)	< 0.0001

### 3.3 Improvement in survival limited to M1b and M1c patients

**FIGURE 3:**
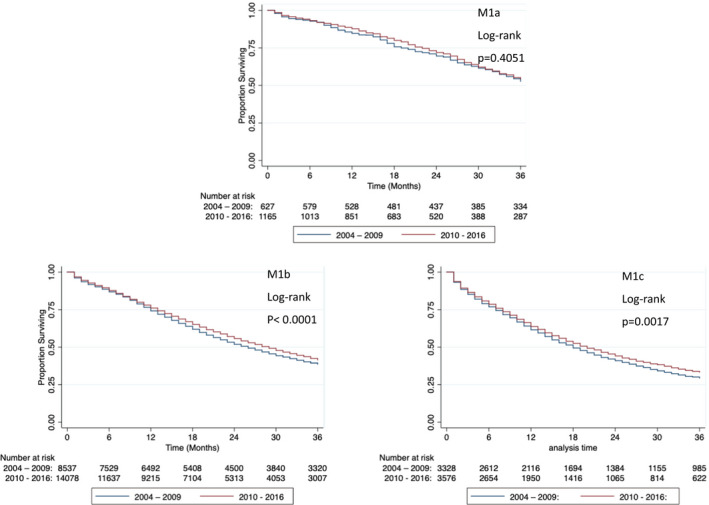
Three‐year overall survival of patients with M1a, M1b, and M1c prostate cancer in 2004–2009 and 2010–2016. M1a overall survival did not change from 2004–2009 to 2010–2016, while M1b and M1c overall survival improved from 2004–2009 to 2010–2016

**FIGURE 4:**
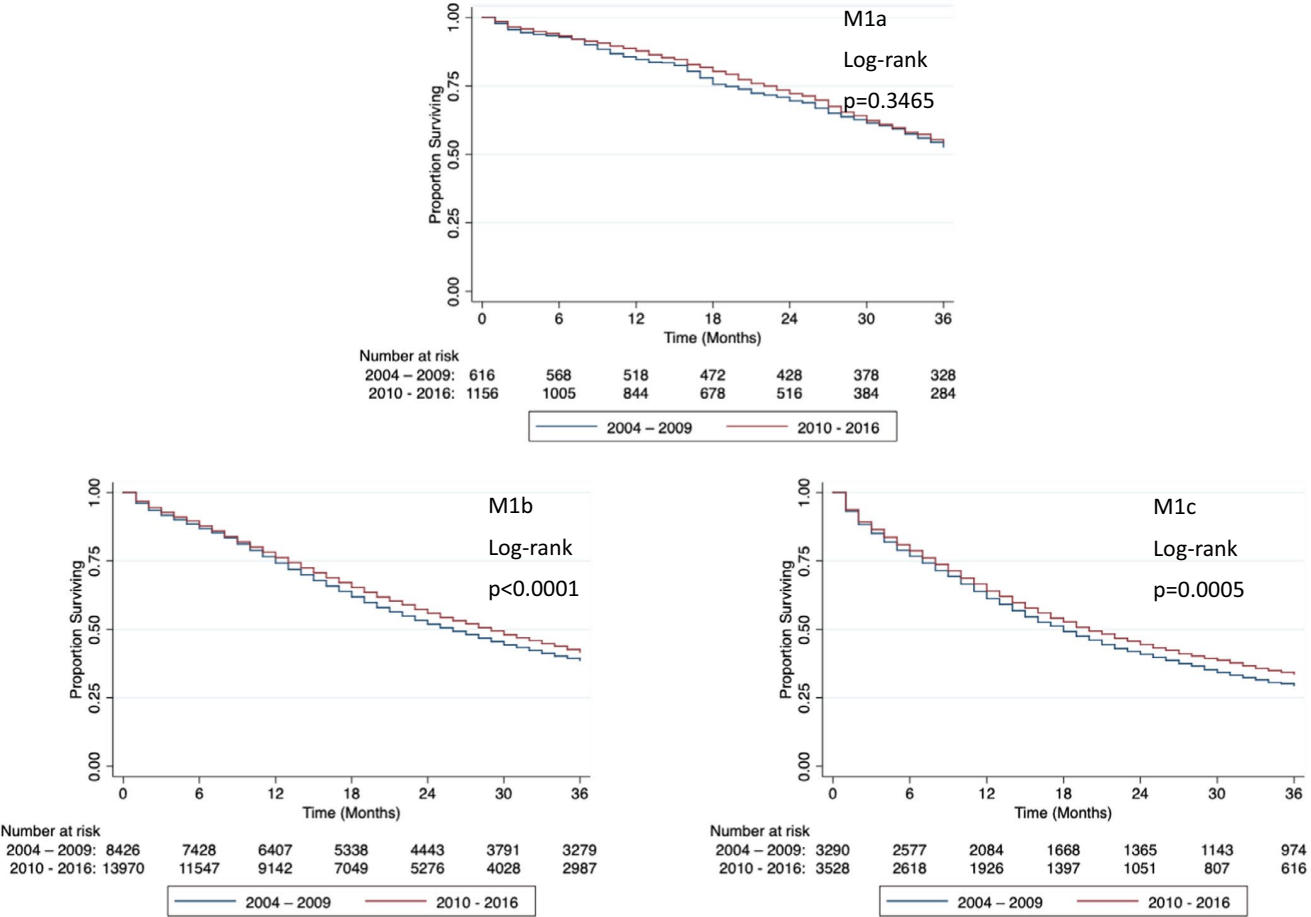
Three‐year prostate cancer‐specific survival of patients with M1a, M1b, and M1c prostate cancer in 2004–2009 and 2010–2016. M1a and M1c prostate cancer‐specific survival did not change from 2004–2009 to 2010–2016, while M1b prostate cancer‐specific survival improved from 2004–2009 to 2010–2016

While the stage is not provided in era 1, the data were available for 12492 and 18819 men in era 2 and 3, respectively (Table 4). When 3‐year OS was stratified by stage between era 2 and 3, we found that patients with M1a prostate cancer did not benefit (Figure 3, *p *= 0.4051, log‐rank test). However, men with M1b and M1c demonstrated improvements in 3‐year OS (Figure 3, *p *< 0.0001 and *p *= 0.0017, respectively, log‐rank test). Patients with M1b prostate cancer saw an increase in median OS from 26 months in era 2 to 29 months in era 3, while those with M1c prostate cancer had a more modest increase in median OS from 18 to 20 months across the same time periods (Table 5). Similarly, 3‐year PCSS did not improve between the two eras for men with M1a prostate cancer (Figure 4, *p *= 0.3465, log‐rank test). As for M1b and M1c patients, increase in 3‐year PCSS was again seen in 2010–2016 (Figure 4, *p *< 0.0001 and *p *= 0.0005, log‐rank test, respectively). Between 2004–2009 and 2010–2016, median PCSS increased for men with M1b disease from 26 to 29 months (Table 5).

**TABLE 4:** Characteristics of patients with metastatic prostate cancer by stage

	n (%), 2004–2009	n (%), 2010–2016	*p*‐value
M1a			
Sample size	627	1,165	
Median age (years)	69 (40–97)	68 (40–95)	0.104
Race/ethnicity			0.399
Non‐Hispanic White	422 (67.30%)	733 (62.92%)	
Non‐Hispanic Black	107 (17.07%)	208 (17.85%)	
Hispanic	70 (11.16%)	150 (12.88%)	
Non‐Hispanic Asian/PI	21 (3.35%)	53 (4.55%)	
Non‐Hispanic American Indian/Alaska Native	4 (0.64%)	10 (0.86%)	
Non‐Hispanic Unknown Race	3 (0.48%)	11 (0.94%)	
Local treatment			0.351
RP and/or XRT	31 (4.94%)	70 (6.01%)	
No local therapy	596 (95.06%)	1,095 (93.99%)	
M1b			
Sample size	8,537	14,078	
Median age (years)	72 (35–99)	71 (34–99)	< 0.001
Race/ethnicity			0.011
Non‐Hispanic White	5,483 (64.23%)	9,013 (64.02%)	
Non‐Hispanic Black	1,517 (17.77%)	2,438 (17.32%)	
Hispanic	939 (11.00%)	1,627 (11.56%)	
Non‐Hispanic Asian/PI	524 (6.14%)	818 (5.81%)	
Non‐Hispanic American Indian/Alaska Native	50 (0.59%)	99 (0.70%)	
Non‐Hispanic Unknown Race	24 (0.28%)	83 (0.59%)	
Local treatment			0.370
RP and/or XRT	203 (2.38%)	309 (2.19%)	
No local therapy	8,334 (97.62%)	13,769 (97.81%)	
M1c			
Sample size	3,328	3,576	
Median age (years)	72 (29–99)	70 (39–99)	< 0.001
Race/ethnicity			0.088
Non‐Hispanic White	2,095 (62.95%)	2,139 (59.82%)	
Non‐Hispanic Black	640 (19.23%)	718 (20.08%)	
Hispanic	380 (11.42%)	465 (13.00%)	
Non‐Hispanic Asian/PI	187 (5.62%)	216 (6.04%)	
Non‐Hispanic American Indian/Alaska Native	19 (0.57%)	24 (0.67%)	
Non‐Hispanic Unknown Race	7 (0.21%)	14 (0.39%)	
Local treatment			0.222
RP and/or XRT	66 (1.98%)	57 (1.59%)	
No local therapy	3,262 (98.02%)	3,519 (98.41%)	

**TABLE 5:** Median overall and PCa‐specific survival by stage and era

Stage	Era	Median Overall Survival (95% CI) (months)	Median PCa‐Specific Survival (95% CI) (months)
M1a	2004–2009	40 (36–45)	40 (36–45)
	2010–2016	40 (37–47)	41 (37–48)
M1b	2004–2009	26 (25–27)	26 (25–27)
	2010–2016	29 (28–30)	29 (28–30)
M1c	2004–2009	18 (17–19)	18 (17–19)
	2010–2016	20 (19–21)	20 (19–22)

Using respective era 2 outcomes as referents, Cox proportional hazards model demonstrated that unadjusted and adjusted OS increased from era 2 to era 3 in men with M1b and M1c diagnosis (adjusted HR 0.9309, 95% CI 0.8973–0.9657, *p *< 0.0001 and adjusted HR 0.9306 95% CI 0.8759–0.9886, *p *= 0.0197, respectively) (Table 6). Likewise, concerning PCSS, only those with M1b and M1c prostate cancer demonstrated an increased survival from 2004–2009 to 2010–2016 (adjusted HR 0.9263, 95% CI 0.8927–0.9612, *p *< 0.0001 and adjusted HR 0.9217, 95% CI 0.8671–0.9797, *p *= 0.0088, respectively) (Table 6).

**TABLE 6:** Cox proportional hazards model of overall survival and PCa‐specific survival for metastatic prostate cancer from 2004–2009 to 2010–2016 by stage

Survival	Stage	HR (95% CI)	*p*‐value	Adjusted HR^a^ (95% CI)	*p*‐value
Overall Survival (2004–2009 versus 2010–2016)	M1a	0.9369894 (0.8027662–1.093655)	0.4093	0.9671552 (0.828782–1.129318)	0.6728
	M1b	0.9169056 (0.8838844–0.9511603)	< 0.0001	0.9308705 (0.8973085–0.9656877)	0.0001
	M1c	0.9094882 (0.8561905–0.9661037)	0.0021	0.9305625 (0.8759341–0.9885979)	0.0197
Prostate cancer‐Specific Survival n(2004–2009 versus 2010–2016)	M1a	0.9284632 (0.7944105–1.085137)	0.3508	0.9622792 (0.822893–1.125275)	0.6301
	M1b	0.9116809 (0.8786209–0.9459848)	< 0.0001	0.9262938 (0.8926658–0.9611887)	< 0.0001
	M1c	0.8992394 (0.8461016–0.9557143)	0.0006	0.9216776 (0.8671141–0.9796746)	0.0088

^a^ Adjusted for age, race/ethnicity, and treatment.

## 4 DISCUSSION

In the present study, we have investigated survival in the SAT era in men diagnosed with metastatic prostate cancer using the SEER database. Our study corroborated the findings of a previous report that found no improvements in OS and PCSS in patients with metastatic prostate cancer between 2000–2003 and 2004–2009 despite the approval of docetaxel in 2004.^11^ We did find, however, a modest but statistically significant increase in OS and PCSS of 4 months, between 2004–2009 and 2010–2016. A previous study found a similar increase in PCSS but slightly lower improvement in OS of 3 months between 2004–2008 and 2009–2014,^14^ which when taken with our observations, suggest further improvement in survival in 2015–2016. Overall, our observations suggest that there has been only a marginal improvement in treating metastatic prostate cancer in the modern SAT era.

Although the first agent shown to be effective in treating metastatic CRPC was docetaxel in 2004,^6^ no survival change was noted in this study until the 2010–2016 era. These findings suggest that the approval of additional drugs since 2010 may have contributed to an improved survival not found between 2000–2003 and 2004–2009. For example, first approved in 2010, cabazitaxel was shown to improve the median survival from 12.7 months in patients given the standard mitoxantrone to 15.1 months in patients given cabazitaxel.^15^ More importantly, treatment with cabazitaxel plus prednisone improved overall survival in patients whose disease had progressed during or after docetaxel‐based therapy. Likewise, additional therapies introduced since 2010 such as sipuleucel‐T, abiraterone, and enzalutamide were shown to increase median survival by 4.1, 3.9, and 4.8 months, respectively, in men with metastatic CRPC.^16^, ^17^, ^18^, ^19^ Our finding that median OS increased by 4 months from 2004–2009 to 2010–2016 corroborates the results of the aforementioned clinical trials.

It should be noted that there are alternative explanations for our observation concerning the recent improvement in survival among men diagnosed with metastatic prostate cancer. For example, it is entirely possible that the increased survival in the modern era for M1 prostate cancer patients may be due to increased acceptance and utilization of docetaxel since its approval in 2004. In its phase 3 study, TAX 327, the median survival for patients with metastatic prostate cancer improved from 16.5 months in the mitoxantrone group to 18.9 months in patients given docetaxel every 3 weeks and 17.4 months in patients given weekly docetaxel.^6^ However, given the relatively rapid progression of M1 disease, such delayed benefit of docetaxel is unlikely. A more probable explanation is that docetaxel may not have been embraced readily by treating physicians and patients. Indeed, a recent study has suggested that nearly half of men diagnosed with metastatic prostate cancer are not treated or treated with androgen deprivation therapy only.^20^

Despite numerous advances in treatment including second‐line anti‐androgens (abiraterone and enzalutamide) between 2010 and 2016, it is noteworthy that median OS improved by mere 4 months. Given that such gain is over two decades, the magnitude of the improvement in outcomes is surprisingly small. This finding may be explained in part by previous studies, which report shared resistance between drugs such as abiraterone and enzalutamide. Possible mechanisms include the presence of androgen‐receptor splice variant 7 messenger RNA (AR‐V7) and induction of glucocorticoid receptor expression.^21^, ^22^ Another study found that patients receiving abiraterone before docetaxel were less likely to respond to docetaxel and achieve a PSA response than patients who had not received abiraterone, suggesting cross‐resistance between the two drugs.^23^ Collectively, these published data suggest that the effect of these drugs in prolonging OS and PCSS among patients with metastatic prostate cancer is not additive. In this regard, more intense initial therapies have been endorsed recently,^12^, ^13^ and new second‐line antiandrogens and PARP inhibitors are now available.^8^, ^9^, ^10^ As such, analysis in 3–5 years is necessary to assess whether these newer agents are additive or have overlapping resistance mechanisms with the current standard of care. Additionally, previous studies have reported that the U.S. Preventive Services Task Force's recommendation against PSA screening in 2012 may have contributed to a 25% increase in the rate of newly diagnosed metastatic prostate cancer from 2004 to 2014.^24^ This increase may have worsened some patients’ staging at presentation and further curtailed the survival improvement in mPCa during the SAT era. Notwithstanding, more investigations should be focused on identifying and targeting novel pathways that are outside the conventional androgen signaling pathways to make a major improvement in outcomes in men with metastatic prostate cancer.

When factors associated with improved survival were assessed, local therapy status was associated with the best outcome (adjusted HR 0.3741, 95% CI 0.3277–0.4272, *p *< 0.0001). This observation is consistent with previous reports that confirmed the association between local therapy (surgery or radiation) and longer survival.^25^ More recently, subgroup analyses of prospective studies suggested the benefit of local radiotherapy in men with a low volume metastatic prostate cancer.^26^, ^27^ Currently, large‐scale prospective studies such as SIMCAP (NCT03456843) and SWOG 1802 (NCT03678025) are underway to further assess the role of surgery and/or radiation in men who present with M1 prostate cancer.^28^

Notably, among racial/ethnic groups, Non‐Hispanic Black patients with metastatic prostate cancer had decreased OS and PCCS compared to Non‐Hispanic White patients (adjusted HR 1.0651, 95% CI 1.0288–1.1027, *p *= 0.0004 and adjusted HR 1.0652, 95% CI 1.0287–1.1029, *p *= 0.0004, respectively). Given the recent finding that the survival disparity between White and Black patients was no longer observed after the U.S. Preventive Services Task Force recommended against PSA screening in May 2012,^29^ the current observation suggests that there may be different rates of treatment among Black patients relative to White patients with M1 prostate cancer. Indeed, it has been shown that after adjusting for risk factors such as age, PSA, and sites of metastases, Black patients with metastatic castration‐resistant prostate cancer who were treated in phase III clinical trials with docetaxel and prednisone (DP) or treatments including DP actually reported increased overall survival relative to White patients.^30^ These observations collectively suggest that fewer Black patients with metastatic prostate cancer may be receiving effective treatment regimens compared to White patients. Further analysis is underway to test this hypothesis.

Further stratification of the data by M stage found that the median OS and PCSS increased only in patients with M1b or M1c prostate cancer. Such observation may be a statistical artifact as the sample size of M1a was relatively small (627 in era 2 and 1165 in era 3). Alternatively, potential explanations include a different treatment approach based on stage. For example, the use of SAT may be higher in men with high metastatic burden. However, given that men with prostate cancer die from widely metastatic disease, patients with M1a will likely be treated with the newer agents at some point during disease progression. A more provocative hypothesis is that the biology of M1a prostate cancer may be fundamentally different from that of M1b and M1c diseases. Given that the tumor microenvironment plays a critical role in tumor biology,^31^, ^32^ a more detailed analysis of additional databases combined with basic laboratory studies is necessary to test this concept.

The strength of our current investigation is that the real‐world data with a very large sample size have been analyzed. While previous studies have examined survival improvements in OS and PCSS of mPCa patients over the last few decades, none have conducted survival analyses stratified by M stage and reported a survival benefit limited to patients with bone and visceral metastasis to our knowledge. Notwithstanding, the limitation is that the study is inherently retrospective. Another limitation is that we do not know what if any second‐line treatments these men received. Indeed, many men in this cohort may not have access to these typically expensive medications. Finally, the adoption of new treatments cannot be performed using SEER only. As such, our study should be considered a hypothesis‐generating study.

## 5 CONCLUSIONS

Since 2000–2003, there has been a modest improvement in survival in patients who are diagnosed with metastatic prostate cancer, with increases in median OS and PCSS of 4 months, respectively. Interestingly, the benefit in survival outcome was observed only in M1b and M1c prostate cancer patients.

## CONFLICT OF INTEREST

All authors have no significant conflict of interest with the content of this study.

## ETHICS STATEMENT

This study did not require the approval of an Institutional Review Board (IRB), because the Surveillance, Epidemiology, and End Results Program (SEER) database is a publicly available, de‐identified database.

## Data Availability

This study used data from the Surveillance, Epidemiology, and End Results Program (SEER) database, which is publicly available at https://seer.cancer.gov/data/.
